# Lipoprotein Subfractions Associated with Endothelial Function in Previously Healthy Subjects with Newly Diagnosed Sleep Apnea—A Pilot Study

**DOI:** 10.3390/life13020441

**Published:** 2023-02-04

**Authors:** Alzbeta Hluchanova, Branislav Kollar, Katarina Klobucnikova, Miroslava Hardonova, Michal Poddany, Ingrid Zitnanova, Monika Dvorakova, Katarina Konarikova, Miroslav Tedla, Milan Urik, Pavel Klail, Petr Skopek, Peter Turcani, Pavel Siarnik

**Affiliations:** 1 Department of Neurology, University Hospital Bratislava, 85107 Bratislava, Slovakia; 2 1st Department of Neurology, Faculty of Medicine, Comenius University, University Hospital, 81499 Bratislava, Slovakia; 3 Department of Neurology, General Hospital, 03123 Liptovsky Mikulas, Slovakia; 4 Institute of Medical Chemistry, Biochemistry and Clinical Biochemistry, Faculty of Medicine, Comenius University, 81499 Bratislava, Slovakia; 5 Department of ENT and HNS, Faculty of Medicine, Comenius University, University Hospital, 81499 Bratislava, Slovakia; 6 Institute of Cancer and Genomic Sciences, University of Birmingham, Birmingham B15 2SQ, UK; 7 Department of Pediatric Otorhinolaryngology, University Hospital Brno, 61300 Brno, Czech Republic; 8 Faculty of Medicine, Masaryk University, 62500 Brno, Czech Republic; 9 Department of Otorhinolaryngology, University Hospital in Pilsen, Faculty of Medicine, Charles University, 11000 Pilsen, Czech Republic

**Keywords:** lipoprotein subfractions, obstructive sleep apnea, polysomnography

## Abstract

Background: Obstructive sleep apnea (OSA) activates several pathophysiological mechanisms which can lead to the development of vascular diseases. Endothelial dysfunction (ED) is an initial step in the development of atherosclerosis. The association between ED and OSA has been described in several studies, even in previously healthy subjects. High-density lipoproteins (HDL) were generally considered to be atheroprotective, and low-density lipoprotein (LDL) to be an atherogenic component of lipoproteins. However, recent findings suggest a pro-atherogenic role of small HDL subfractions (8–10) and LDL subfractions (3–7). This study aimed to evaluate the relationship between endothelial function and lipid subfractions in previously healthy OSA subjects. Material and Methods: We prospectively enrolled 205 subjects with sleep monitoring. Plasma levels of triacylglycerols, total cholesterol, LDL, HDL, and their subfractions were assessed. Endothelial function was determined using peripheral arterial tonometry, and reperfusion hyperemia index (RHI) was assessed. Results: Plasma levels of small and intermediate HDL subfractions have statistically significant pro-atherogenic correlations with endothelial function (*p* = 0.015 and *p* = 0.019). In other lipoprotein levels, no other significant correlation was found with RHI. In stepwise multiple linear regression analysis, small HDL (beta = −0.507, *p* = 0.032) was the only significant contributor in the model predicting RHI. Conclusions: In our studied sample, a pro-atherogenic role of small HDL subfractions in previously healthy subjects with moderate-to-severe OSA was proven.

## 1. Introduction

Obstructive sleep apnea (OSA) is a common disorder that occurs in approximately one-quarter of adults. The prevalence of OSA (apnea/hypopnea index (AHI) over 5) is about 24% in men and 9% in women [1]. Unrefreshing sleep with excessive sleepiness is the most common presenting symptom of OSA. Patients experience snoring as well as awakenings accompanied by gasping or choking. Effective therapeutic approaches include weight loss, positive airway pressure, oral appliances, surgical modification of the pharyngeal soft tissues or facial skeleton, and hypoglossal nerve stimulation in selected cases [2]. OSA is characterized by repeated partial or complete obstructions in the upper airways, leading to large changes in intrathoracic pressure, consequent hemodynamic changes, chronic intermittent hypoxia, and sleep fragmentation [3]. Intermittent hypoxemia with concomitant hypercapnia activates the sympathetic nervous system and contributes to elevation of blood pressure. Repetitive respiratory events increase reactive oxygen species, which may also contribute to vascular disease [3]. OSA activates several other pathophysiological mechanisms which can lead to the development of vascular diseases including endothelial dysfunction, systemic inflammatory response, and impaired glucose and lipid metabolism [4,5]. Endothelial dysfunction is an important underlying pathophysiological mechanism of atherogenesis that occurs at the early stages of vascular diseases and is associated with the occurrence of vascular events in the future [6,7]. Multiple mechanisms underlying OSA, including oxidative stress and systemic inflammation, are also important mechanisms in the pathogenesis of decreased nitric oxide bioavailability and subsequent endothelial dysfunction [8]. Endothelial dysfunction can be assessed by various methods, including peripheral arterial tonometry (PAT). The reperfusion hyperemia index (RHI) measured by PAT is a validated marker of endothelial function and a predictor of future vascular events [9,10,11]. Endothelial dysfunction is defined by RHI as lower than 1.67. Endothelial dysfunction is present also in previously healthy subjects with newly diagnosed OSA, and OSA is associated with the severity-dependent impairment of endothelial function assessed by RHI in both adults and children [12,13,14]. Dyslipidemia, defined as an excessive increase in total cholesterol (TC) or triacylglycerols (TAG), with or without a concomitant decrease in high-density lipoproteins (HDL), leads to the acceleration of the atherosclerotic process in predisposed individuals and is also one of the most important risk factors for vascular disease [15]. Along with increased sympathetic nervous system activity, oxidative stress, systemic inflammation, subsequent hypertension and glucose metabolism impairment [16], dyslipidemia is one of the possible mechanisms linking OSA with increased vascular morbidity [17]. The mechanisms of dyslipidemia induced by OSA include the up-regulation of lipoprotein biosynthesis, increased lipolysis, impaired clearance of lipoproteins, lipoprotein peroxidation, and HDL dysfunction [18]; this may represent a pathway by which the increase in cardiovascular risk is mediated [19]. There is also increasing evidence that small dense LDL and HDL subfractions have pro-atherogenic properties [20,21]. This study aimed to evaluate the relationship between endothelial function and lipid subfractions in previously healthy OSA subjects.

## 2. Materials and Methods

### 2.1. Study Population

A total of 205 patients were included in this prospective monocentric study. The cohort includes patients who were suspected of suffering from OSA and were hospitalized in the sleep laboratory of the 1st Department of Neurology, Comenius University, and University Hospital Bratislava. The study was approved by the Ethics Committee of the Old Town Hospital, University Hospital Bratislava (with reference number 26/2021). All participants signed informed consent before enrollment.

**Figure 1:**
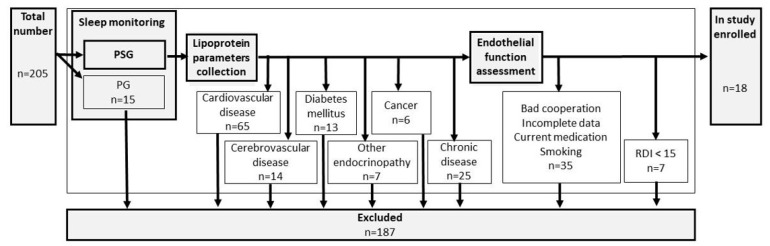
Flowchart of the study.

Inclusion criteria were set up as follows: suspicion of OSA, sleep monitoring by polysomnography, and aged over 18. A detailed search for premorbid diseases was performed and exclusion criteria included: cardiovascular disease, cerebrovascular disease, diabetes mellitus, other endocrinopathies, cancer, or any other chronic diseases. Sleep monitoring by limited polygraphy (PG), bad cooperation, incomplete data, use of any current medication, or smoking belonged to the additional exclusion criteria. For details, see flowchart Figure 1. 

**Figure 2:**
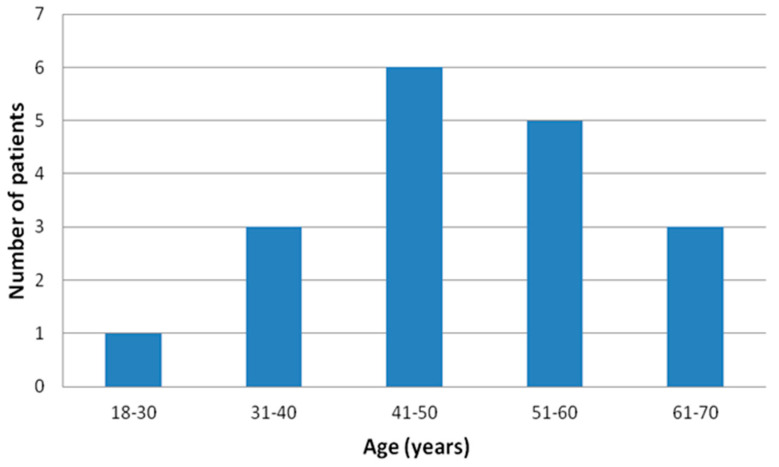
Age distribution of the final enrolled patients’ sample.

Finally, apparently healthy 18 male subjects, the “real-world” population with no previous history of sleep apnea, age 47.9 ± 11.5 (Figure 2), body mass index (BMI) 32.3 ± 3.8 kg/m^2^, investigated in this tertiary hospital from March 2021 to March 2022 were prospectively enrolled.

### 2.2. Methods

Sleep monitoring

All subjects underwent overnight sleep monitoring. In the study, only patients with polysomnography (Alice 6 device, Philips-Respironics, Murrysville, PA, USA) and recorded respiratory disturbance index (RDI) were enrolled. Only subjects with RDI ≥ 15 were included for further assessment. Other recorded indices included oxygen desaturation index (ODI), arousal index, average nocturnal O_2_ saturation, and minimal nocturnal O_2_ saturation. Standardized criteria were used for the scoring of sleep characteristics and respiratory events [22]. 

Monitored variables:

BMI (body mass index)—defined as body weight divided by the square of height; a measure of the degree of obesity.
Apnea—defined as the reduction in airflow ≥90% (or the airflow cessation) lasting >10 s.
Hypopnea—defined as a reduction in airflow ≥30% lasting >10 s with oxygen desaturation ≥3% or arousal.
Respiratory disturbance index (RDI)—defined as an average number of apneas, and hypopneas per 1 h of sleep.
ODI (oxygen desaturation index)—defined as a number of desaturations ≥3% with a duration of >10 s per hour of sleep.
Arousal index—defined as the total number of arousals per hour of sleep.
Average nocturnal O_2_ saturation—defined as the mean O_2_ saturation during sleep.
Minimal nocturnal O_2_ saturation—defined as the lowest single O_2_ saturation seen in the recording.
Time with O_2_ saturation <90% (T90)—defined by the percentage of sleep time below 90% O_2_ saturation.

Lipoprotein parameters

Blood plasma samples were obtained in the morning after polysomnography and after overnight fasting. Blood samples with ethylenediaminetetraacetic acid (EDTA) were collected. Immediately after the collection of plasma samples, levels of TAG, TC, LDL, and HDL were determined in a local certified hospital laboratory with an enzymatic method (Roche Diagnostics, Mannheim, Germany). The quantitative analysis of lipoprotein families and lipoprotein subfractions including very low-density lipoprotein (VLDL), intermediate-density lipoprotein (IDL), and plasma lipoprotein subfractions were analyzed by the Lipoprotein system (Quantimetrix Corp., Redondo Beach, CA, USA) using a polyacrylamide gel electrophoresis [23]. The following subfractions were evaluated: large LDL subfractions 1–2 (which are considered atheroprotective), small dense LDL subfractions 3–7 (which are considered atherogenic), large HDL subfractions 1–3 (which are considered atheroprotective), small dense HDL subfractions 8–10 (which are considered atherogenic), and intermediate HDL subfractions 4–7 (their atherogenic/atheroprotective role remains controversial) [20,21].

Assessment of endothelial function

Endothelial function was assessed by PAT (EndoPAT 2000 device, Itamar Medical Ltd., Caesarea, Israel) as previously described [14]. RHI was calculated as the ratio of the average amplitude of the PAT signal post-to-pre occlusion of the tested arm, normalized to the concurrent signal from the contralateral finger. Calculations were performed using the computer algorithm (software 3.1.2) supplied with the device. RHI value < 1.67 indicated endothelial dysfunction [14,24].

Statistical analysis

Statistical analyses were performed by SPSS ver. 18 (SPSS Inc., Chicago, IL, USA). The results of normally distributed data are expressed as a mean ± standard deviation, and the results of not normally distributed data are expressed as median, interquartile range, minimal and maximal values. Pearson or Spearman correlation coefficients were used to determine the relationships between RHI and the baseline characteristics of the study population. We used stepwise multiple linear regression to create the prediction model and identify the most important contributors to this model. A model with the highest number of significant predictors was chosen. The dependent variable in the model was RHI, independent variables in the model were anthropometric characteristics (age, gender, BMI), sleep characteristics (T90, RDI, ODI, arousal index, average, and minimal nocturnal O_2_ saturation), and lipoprotein levels (TAG, TC, LDL, HDL, VLDL, IDL, large LDL, small LDL, large HDL, intermediate HDL, and small HDL). Each model was assessed for the presence of multicollinearity of included variables. The variance inflation factor (VIF) ≥5 was indicative of multicollinearity. The *p* value < 0.05 was considered statistically significant.

## 3. Results

The average age of participants was 47.9 years, the average BMI value was 32.31 kg/m^2^. All participants were in the category of moderate-to-severe sleep apnea with a mean RDI of 45.7. The average nocturnal O_2_ saturation was 87.8, the average minimal nocturnal O_2_ saturation was 76.2. None of these monitored variables were statistically significantly correlated with RHI. The results of normally distributed data are expressed as a mean ± standard deviation, and the results of not normally distributed data are expressed as median, interquartile range, minimal and maximal values. For details, see Table 1. 

**Figure 3:**
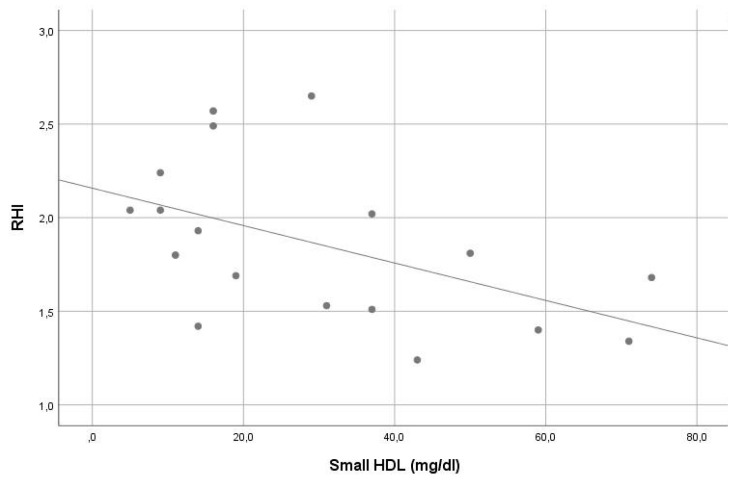
Correlation of small high-density lipoprotein levels with reperfusion hyperemia index (r = −0.561, *p =* 0.015).

**Figure 4:**
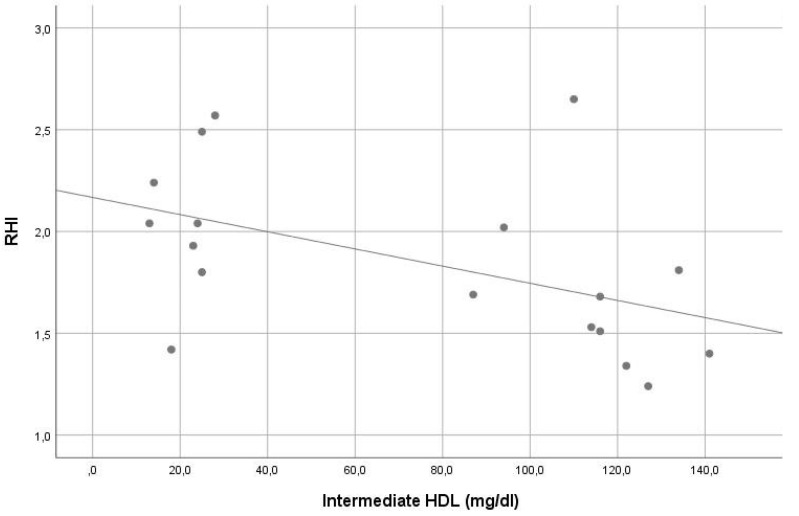
Correlation of intermediate high-density lipoprotein levels with reperfusion hyperemia index (r = −0.548, *p =* 0.019).

In our sample, the average RHI was 1.9. Except for the significant inverse correlation of small HDL and intermediate HDL with RHI (r = −0.561, *p =* 0.015 and r = −0.548, *p =* 0.019, consecutively), no other significant correlation was found between RHI and other lipoprotein levels (see Table 2). In stepwise multiple linear regression analysis, small HDL (beta = −0.507, *p* = 0.032) was the only significant contributor in the model predicting RHI. The VIF of all variables assessed in this model was <5. For details, see Table 1 and Table 2, Figure 3 and Figure 4.

## 4. Discussion

OSA activates several pathophysiological mechanisms that lead to the development of vascular diseases, as discussed in recent studies by Ott et al. in 2017 [25], Kollar et al. in 2021 [21], and many other authors [26,27,28]. The potential underlying pathomechanism linking OSA with the development of vascular diseases include endothelial dysfunction [29,30], activation of the sympathetic nervous system [3], oxidative stress [31], metabolic dysregulation [32], activation of inflammatory processes [29,33], and alteration of the coagulation cascade [29]. Levy et al. in 2009 [34] already described, in agreement with Seiler et al. in 2019 [35], that acceleration of atherogenesis could be one of the most important mechanisms involved in the development of vascular diseases in OSA patients. Endothelial dysfunction as the initial step and key process of atherogenesis was proven, for example by Bonetti et al. in 2003 or Gimbrone et al. in 2016 [36,37]. The association between endothelial dysfunction and OSA has been described in several studies [12,13,14] and was found also in patients with OSA who are not treated for any other diseases, like the findings presented in the work of Ip et al. in 2004 or Siarnik et al. in 2014 [14,38]. 

Our study was based on a strict exclusion process, and the final sample of apparently healthy subjects of our “real-world” population with no previous history of sleep apnea had only 18 responders; at the time, this was a pilot project on the topic of the possible association of lipoprotein subfractions with endothelial function in previously healthy individuals (with newly diagnosed sleep apnea). Juházs in 2014 [17] suggested dyslipidemia as one of the possible mechanisms linking OSA with increased vascular morbidity. The same opinion was presented by Helkin et al. in 2016 [17,39]. LDL is generally considered to be atherogenic and HDL to be atheroprotective. However, there are increasing data that small LDL (3–7) and small HDL (8–10) subfractions have atherogenic properties [20,21,40]. Our results suggest a pro-atherogenic role of small HDL subfractions in previously healthy subjects with moderate-to-severe OSA. Small HDL (beta = −0.507, *p* = 0.032) was the only significant contributor in the model predicting RHI—a measure of endothelial function, in stepwise multiple linear regression analysis (see Results). The pro-atherogenic lipoprotein phenotype characterized by increased levels of atherogenic lipoprotein subfractions and reduced levels of atheroprotective subfractions was found in individuals with OSA. In this population, significantly lower levels of atheroprotective LDL1 and large HDL subfractions were detected as well as significantly higher levels of atherogenic small dense LDL 3–7 subfractions [21]. Our results are consistent with the findings of previously mentioned studies [17,41,42,43,44,45]. Among our previously healthy patients with newly diagnosed moderate-to-severe OSA, small HDL was the only significant predictor of RHI, suggesting a pro-atherogenic role of small HDL subfractions in this population. We are not aware of any similar study so far. 

Although only previously healthy subjects were enrolled and the use of any current medication or smoking belonged to additional exclusion criteria, the co-administration of other supplements as antioxidants and anti-inflammatory substances with beneficial effect on vascular status were not taken in consideration. This fact limits the findings of the current study. For example, the use of micronized purified flavonoid fraction of Rutaceae aurantiae in type 2 diabetic patients proved to reduce the risk of cardiovascular disease [46]. In another study, omega-3 proved its beneficial effect on serum lipid profile and oxidative stress [47]. For endothelial dysfunction, it was found that ramipril [48] as well as febuxostat have a direct ameliorating effect on inflammation and oxidative stress in patients with endothelial dysfunction, which is an important risk factor for cardiovascular diseases [49,50]. Similarly, the oral cholecalciferol effect on vascular calcification and 25(OH)D levels was investigated, which significantly increased serum levels of 25(OH)D and fetuin-A [51]. 

The enrollment of previously healthy subjects with newly diagnosed sleep apnea is the strength of the current study as it limits the effect of other possible pro-atherogenic confounders. However, a strict exclusion process leads to a small sample of respondents. This pilot study shows that in future large multicenter prospective studies with detailed blood pressure assessment, glycemia testing, a search for anthropometric parameters and physical activity measures, as well as a search for the effect of CPAP on lipoprotein subfractions and endothelial function measures should be beneficial. The effect of lifestyle interventions on LDL and HDL subfractions is known from the results of previous studies [52,53,54,55].

## 5. Conclusions

In our studied sample of previously healthy subjects with moderate-to-severe OSA, the plasma levels of small and intermediate HDL subfractions have statistically significant pro-atherogenic correlations with endothelial function (*p* = 0.015 and *p* = 0.019), but after stepwise multiple linear regression analysis, we conclude that a pro-atherogenic role was proven only for small HDL. Small HDL was the only significant contributor in the model predicting RHI (beta = −0.507, *p* = 0.032). We are not aware of any similar findings so far. No other significant lipoprotein level correlation was found.

## Figures and Tables

**Table 1:** Sleep monitoring indices and their correlations with reperfusion hyperemia index.

N = 18	Mean	Median	Standard Deviation	Interquartile Range	Minimal Value	Maximal Value	Correlation		Regression	
							r	*p*	Beta	*p*
Age (years)	47.9	49.0	11.5	18	26	66	0.087	0.732	0.139	0.539
BMI (kg/m^2^)	32.3	31.9	3.8	6.5	26.6	39.6	−0.278	0.264	−0.048	0.842
RDI (n/h)	45.7	43.6	20.5	35.6	15.8	84.6	−0.088	0.729	−0.092	0.685
ODI (n/h)	39.9	36.8	19.4	29.7	12.8	85.7	−0.061	0.810	−0.069	0.760
Arousal index (n/h)	25.9	24.8	14.0	21.8	7.9	53.7	0.034	0.893	−0.071	0.752
Average nocturnal O_2_ sat. (%)	87.8	88.0	3.3	6	81	92	0.283	0.256	0.298	0.176
Minimal nocturnal O_2_ sat. (%)	76.2	77.5	8.7	10	55	87	0.284	0.254	0.336	0.124
T90 (%)	11.3	7.0	14.1	11.0	0.2	47.4	−0.425	0.079	−0.192	0.393

BMI: body mass index, RDI: respiratory disturbance index, ODI: oxygen desaturation index, sat.: saturation, T90: Time with O_2_ saturation <90%.

**Table 2:** Baseline laboratory characteristics and correlations of the reperfusion hyperemia index with variables.

N = 18	Mean	Median	Standard Deviation	Interquartile Range	Minimal Value	Maximal Value	Correlation		Regression	
							r	*p*	Beta	*p*
TC (mmol/L)	5.3	5.5	0.8	1.3	3.7	6.4	−0.116	0.646	−0.016	0.947
LDL (mmol/L)	4.0	4.1	0.7	1.0	2.6	5.2	−0.126	0.618	−0.080	0.724
HDL (mmol/L)	1.2	1.1	0.3	0.4	0.7	1.8	−0.086	0.735	−0.031	0.893
TAG (mmol/L)	2.1	1.8	0.9	0.9	1.0	4.4	0.218	0.385	0.279	0.276
VLDL (mg/dL)	56.6	60.5	14.0	23.0	32.0	85.0	0.146	0.564	0.152	0.502
IDL (mg/dL)	45.9	45.0	14.3	27.0	26.0	72.0	−0.281	0.259	−0.106	0.643
Large LDL (mg/dL)	48.3	48.5	12.5	13.8	21.0	70.0	−0.090	0.722	−0.141	0.535
Small LDL (mg/dL)	10.2	6.0	10.1	17.3	0	29.0	0.278	0.264	0.237	0.286
Large HDL (mg/dL)	27.8	22.5	23.8	35.25	1.0	81.0	−0.415	0.087	−0.249	0.309
Intermediate HDL (mg/dL)	73.0	90.5	50.1	93.75	13.0	141.0	−0.548	***0.019***	−0.224	0.602
Small HDL (mg/dL)	30.2	24.0	21.7	31.5	5.0	74.0	−0.561	***0.015***	***−0.507***	***0.032***
RHI	1.9	1.8	0.4	0.6	1.2	2.7	-	-	-	-

TC: cholesterol, TAG: triacylglycerols, HDL: high-density lipoprotein cholesterol, LDL: low-density lipoprotein cholesterol, VLDL: very low-density lipoprotein, IDL: intermediate-density lipoprotein, RHI: reperfusion hyperemia index. Significant associations in bold.

## Data Availability

The data presented in this study are available on request from the corresponding author.

## Abbreviations

BMI	body mass index
CPAP	continuous positive airway pressure
EDTA	ethylenediaminetetraacetic acid
HDL	high-density lipoprotein cholesterol
IDL	intermediate-density lipoprotein
LDL	low-density lipoprotein cholesterol
ODI	oxygen desaturation index
OSA	obstructive sleep apnea
PAT	peripheral arterial tonometry
PG	polygraphy
PSG	polysomnography
RDI	respiratory disturbance index
RERAs	respiratory effort-related arousal
RHI	reactive hyperemia index
VLDL	very low-density lipoprotein
